# Senescent Changes in Sensitivity to Binaural Temporal Fine Structure

**DOI:** 10.1177/2331216518788224

**Published:** 2018-07-20

**Authors:** Christian Füllgrabe, Aleksander P. Sęk, Brian C. J. Moore

**Affiliations:** 1 Medical Research Council Institute of Hearing Research, School of Medicine, University of Nottingham, UK; 2 Institute of Acoustics, Faculty of Physics, Adam Mickiewicz University, Poznan, Poland; 3 Department of Psychology, University of Cambridge, UK

**Keywords:** binaural hearing, aging, interaural phase, suprathreshold processing, older listeners

## Abstract

Differences in the temporal fine structure (TFS) of sounds at the two ears are used for sound localization and for the perceptual analysis of complex auditory scenes. The ability to process this binaural TFS information is poorer for older than for younger participants, and this may contribute to age-related declines in the ability to understand speech in noisy situations. However, it is unclear how sensitivity to binaural TFS changes across the older age range. This article presents data for a test of binaural sensitivity to TFS, the “TFS-adaptive frequency” (AF) test, for 118 listeners aged 60 to 96 years with normal or near-normal low-frequency hearing, but a variety of patterns of hearing loss at higher frequencies. TFS-AF scores were significantly lower (i.e., poorer) than those for young adults. On average, scores decreased by about 162 Hz for each 10-year increase in age over the range 60 to 85 years. Individual variability increased with increasing age. Scores also declined as low-frequency audiometric thresholds worsened. The results illustrate the range of scores that can be obtained as a function of age and may be useful for the diagnosis and management of age-related hearing difficulties.

## Introduction

The ability to process speech, which has an important influence on the social integration (Mick, Kawachi, & Lin, 2014; Strawbridge, Wallhagen, Shema, & Kaplan, 2000) and the psychological and cognitive well-being of a person (Gopinath et al., 2009; Lin et al., 2011), declines with increasing age (Bergman et al., 1976; Pronk et al., 2013). Hearing sensitivity, generally assessed by pure-tone audiometry, also worsens across the lifespan (Bunch, 1929; Cruickshanks et al., 1998), and the resulting reduced audibility is known to be associated with impaired speech intelligibility (Delk, Glorig, Quiggle, & Summerfield, 1957; Harris, Haines, & Myers, 1956). A causal relationship between speech perception and audibility is supported by studies showing that (a) experimentally reducing spectral energy in frequency bands that contain important speech information results in lower speech intelligibility (Vickers, Robinson, Füllgrabe, Baer, & Moore, 2009) and (b) providing frequency-specific amplification to hearing-impaired (HI) listeners improves speech identification in quiet and in noise (Shanks, Wilson, Larson, & Williams, 2002). Not surprisingly, pure-tone audiometry has been considered the clinical gold standard for the assessment of hearing health and prediction of hearing handicap. Hearing aids (HAs), which provide amplification on the basis of the patient’s audiogram, currently represent the most common form of auditory rehabilitation for speech-perception difficulties.

Although HAs can improve the ability to hear soft speech, they usually do not restore speech intelligibility to “normal” (Pavlovic, 1984). A large proportion of HI listeners fitted with HAs never or rarely use them (Knudsen, Oberg, Nielsen, Naylor, & Kramer, 2010), presumably because they are perceived as not providing sufficient benefit. Also, speech-perception deficits for older listeners are observed even when their audiometric thresholds are matched to those of young normal-hearing (YNH) listeners (Füllgrabe, Moore, & Stone, 2015). This strongly suggests that factors “beyond the audiogram” need to be considered in the diagnosis and management of age-related hearing difficulties (Jerger, 1992; Kricos, 2006; Musiek, Shinn, Chermak, & Bamiou, 2017).

It is often assumed that the audiogram primarily reflects processes involved in the transduction and cochlear amplification of sounds via the “active mechanism” (Moore & Glasberg, 2004). Deficits in speech perception for older listeners with normal audiograms probably reflect effects of age on biochemical, physiological, and morphological processes other than the active mechanism in the cochlea. Age-related changes occur from the auditory periphery (Sergeyenko, Lall, Liberman, & Kujawa, 2013) to more central portions of the auditory system (Harris & Dubno, 2017), and in the brain in general (Meunier, Stamatakis, & Tyler, 2014). There are large individual differences in some auditory abilities across and within age groups (e.g., Kidd, Watson, & Gygi, 2007; Surprenant & Watson, 2001), and, for some of these abilities, these variations might explain variability in speech perception (Festen & Plomp, 1990; Glasberg & Moore, 1989).

One aspect of suprathreshold processing that has received considerable attention in recent years is the ability to process temporal fine structure (TFS) information. In the cochlea, complex broadband signals, such as speech, are decomposed by the filtering on the basilar membrane (BM) into a series of narrowband signals. The waveform at each place on the BM can be considered as an envelope (ENV) superimposed on a more rapidly oscillating carrier, the TFS. Moore (2014) distinguished between the physical ENV and TFS of the input signal (ENV_p_ and TFS_p_), the ENV and TFS at a given place on the BM (ENV_BM_ and TFS_BM_), and the neural representation of ENV and TFS (ENV_n_ and TFS_n_). TFS_n_ depends on the synchronization of action potentials to individual cycles of TFS_BM_, that is, on phase locking to TFS_BM_. Here, “ENV” and “TFS” are used as generic terms to refer to ENV_BM_ and ENV_n_ on one hand and TFS_BM_ and TFS_n_ on the other hand.

There are several reasons for the increased interest in how the auditory system specifically processes TFS information within monaural and binaural pathways. First, ENV sensitivity worsens only marginally with age (Füllgrabe et al., 2015; Wallaert, Moore, & Lorenzi, 2016) and is not affected (Moore & Glasberg, 2001) or is positively affected by age-related hearing loss (ARHL; due to the loss in cochlear compression; Füllgrabe, Meyer, & Lorenzi, 2003), while both age and ARHL have adverse effects on the processing of TFS information (e.g., Füllgrabe & Moore, 2014; Gallun et al., 2014; Pichora-Fuller & Schneider, 1992; Ross, Fujioka, Tremblay, & Picton, 2007; Santurette & Dau, 2007; for a meta-analysis, see Füllgrabe & Moore, in press). Second, sensitivity to changes in TFS has been shown to be associated with (a) the variability in speech-in-noise (SiN) identification performance observed for YNH (Oberfeld & Klöckner-Nowotny, 2016) and older normal-hearing (ONH; Füllgrabe et al., 2015) listeners, (b) the speech-identification difficulties of unaided (Strelcyk & Dau, 2009) and aided (Lopez-Poveda et al., 2017) HI listeners, and (c) self-reported HA benefit for HI listeners (Perez, McCormack, & Edmonds, 2014). It has been suggested that the ability to use TFS information improves the understanding of speech in the presence of interfering sounds by enhancing the perceptual segregation of the target from the background (Moore, 2008; Stone, Moore, & Füllgrabe, 2011), for example, based on differences in perceived direction (Neher, Lunner, Hopkins, & Moore, 2012) and fundamental frequency (Brokx & Nooteboom, 1982).

The converging evidence for the importance of TFS information for speech perception in everyday listening situations and the finding that increasing age and mild hearing loss can impair the ability to process TFS (Hopkins & Moore, 2007; Ross, Fujioka, et al., 2007) have led to the recent focus on the development of behavioral tests that could be used in large-scale research studies or audiology clinics to assess TFS sensitivity (Sęk & Moore, 2012; Sheft, Risley, & Shafiro, 2012). One test of the binaural processing of TFS is the TFS-low frequency (LF) test (Hopkins & Moore, 2010), in which the task is to distinguish an interaural phase difference (IPD) of ϕ from an IPD of 0° in bursts of pure tones with a fixed frequency. Several studies using this test have shown that performance worsens with increasing age (e.g., Füllgrabe et al., 2015; Moore, Vickers, & Mehta, 2012). However, only a few studies have used the test with large groups of participants (with *N* > 100), and they always tested participants with a wide range of ages, including young adults, and age effects were reported across the entire adult life span (Füllgrabe, 2013; Rönnberg et al., 2016). In studies focussing exclusively on older participants, the sample size was generally much smaller (typically *N* ≤ 40), and the age range investigated was unevenly sampled (e.g., Moore, Glasberg, Stoev, Füllgrabe, & Hopkins, 2012). Hence, data on the effects of age throughout older adulthood on performance of the TFS-LF test are not available.

The TFS-LF test has an important limitation in that a considerable number of older listeners are unable to perform the task, and hence, no graded measure of sensitivity to TFS can be obtained for those listeners. Füllgrabe, Harland, Sęk, and Moore (2017) modified the TFS-LF test to overcome this limitation. In their test, referred to as the TFS-AF test (where AF stands for adaptive frequency), the IPD is fixed and the frequency of the tone is adaptively varied. A similar procedure was used in earlier studies (Grose & Mamo, 2010; Neher, Laugesen, Jensen, & Kragelund, 2011; Ross, Fujioka, et al., 2007; Ross, Tremblay, & Picton, 2007; Santurette & Dau, 2007). The task becomes impossible when the frequency is too high, but the highest frequency at which the task can be performed varies across listeners and provides a measure of binaural sensitivity to TFS. The TFS-AF test has the advantage that all listeners tested in previously published studies could complete the task, independently of their age and hearing status (Füllgrabe et al., 2017; Füllgrabe & Moore, 2017). Also, as for the TFS-LF test, reliable threshold estimates can be obtained relatively quickly (with a single test run typically taking about 5 min) and without practice (Füllgrabe & Moore, 2017).

The aim of the present study was to establish typical changes in performance of the TFS-AF test across older adulthood, using a large cohort of listeners aged above 60 years and with normal or near-normal low-frequency hearing, but with a variety of patterns of hearing loss at higher frequencies. The results were intended to provide reference data for other research studies, and to facilitate interpretation of results obtained for individuals who might be tested in audiology clinics, and thereby help to predict speech-perception performance and guide the selection of HA signal processing for a given individual (Füllgrabe et al., 2017). To characterize our cohort, demographic data were gathered, and two tests of cognitive ability were administered for each participant. This potentially allows comparison of our participants with patients seen in audiology clinics.

## Methods

### General Methodology

Community-dwelling participants, aged 60 years and above, were sought through public advertisements (e.g., in social clubs, doctors’ surgeries, and local newspapers) and from existing participant databases. The study was conducted at two sites in the United Kingdom, the cities of Cambridge and Nottingham, with the aim of recruiting participants from a wide demographic background. Cambridge is a small city (approximately 130,000 inhabitants), influenced by and centered around its historic university and colleges. Cambridge has a much higher than average proportion of people in the highest paid professional, managerial, and administrative jobs. Nottingham is a medium-sized city (approximately 321,500 inhabitants) in the historically (more) industrial Midlands of the United Kingdom.

The study was approved by the Cambridge Research Ethics Committee and the University of Nottingham’s School of Psychology Ethics Committee. Prior to data collection, participants provided informed written consent. Participants received an hourly wage for their services.

### Audiometric and Cognitive Screening and Sociodemographic Characteristics of Participants

All testing was conducted in a sound-attenuating booth. Cognitive assessment was performed using paper-and-pencil materials that were standard for each test. The TFS-AF test used stimuli that were digitally synthesized using a PC, converted to analog form using an external RME babyface soundcard with 24-bit resolution and a sampling rate of 48000 Hz, and presented via Sennheiser HDA200 headphones.

**Figure 1.**
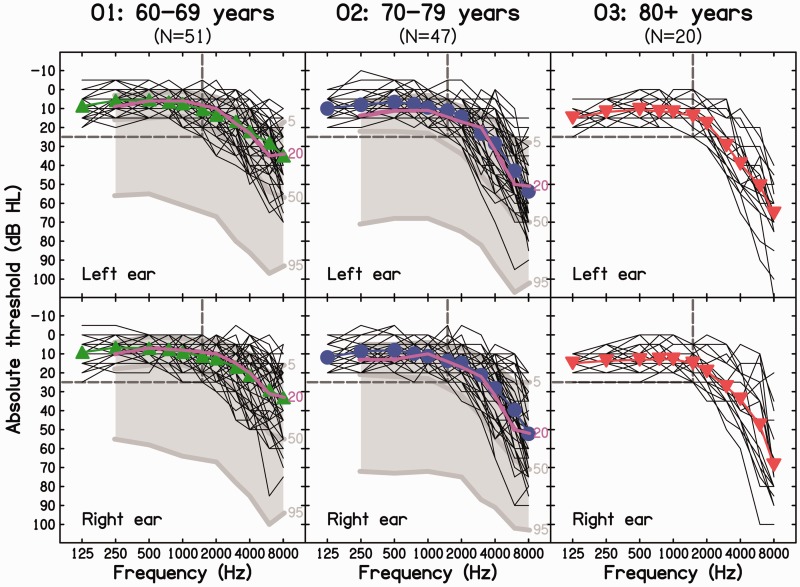
Individual (thin lines) and mean (filled symbols and thick lines) audiometric thresholds (in dB hearing level [HL]) for frequencies between 125 and 8000 Hz for each ear and for three age groups: 60–69 years (left panels, O1), 70–79 years (middle panels, O2), and 80 years and above (right panels, O3). The dashed dark gray lines indicate the audiometric inclusion criteria used in this study. Comparison data (in the form of audiograms corresponding to the 5^th^, 50^th^, and 95^th^ percentiles, pale gray lines) are shown for the population-representative sample from the “National Study of Hearing” conducted by the MRC Institute of Hearing Research. The pink lines in the left and middle panels show the 20^th^ percentile from the same study.

Air-conduction pure-tone audiometric thresholds were assessed following the procedure recommended by the British Society of Audiology (2004) and using standard calibrated audiometric equipment. Thresholds were measured for each ear at octave frequencies from 125 to 8000 Hz, as well as at 750, 1500, 3000, and 6000 Hz. We included participants for whom audiometric thresholds were normal or near-normal (≤25 dB hearing level [HL]) at low frequencies (≤1500 Hz) but whose high-frequency hearing sensitivity varied from normal to moderately to severely impaired. In addition, participants were selected to have a small interaural asymmetry in audiometric thresholds (≤15 dB) for frequencies up to 1500 Hz, which covers the range where discrimination of IPD on the basis of TFS is possible, that is, on average up to about 1300 Hz (Brughera, Dunai, & Hartmann, 2013; Füllgrabe et al., 2017). The individual and mean audiometric thresholds are shown in Figure 1. The data were analyzed based on three, roughly decade-wide, age groups: 60–69, 70–79, and 80+ years. We decided to use three groups so as to have a reasonably large number of participants in each group. For convenience, these groups are denoted O1, O2, and O3, respectively.

On average, audiometric thresholds worsened progressively with increasing frequency above 1500 Hz and with increasing age. For comparison, mean audiometric thresholds for decade-wide age groups from a UK population-representative sample, reported in the “National Study of Hearing” conducted by the MRC Institute of Hearing Research (Davis, 1995), are shown for each ear for Groups O1 and O2 (gray shaded areas and lines without symbols); reference data for Group O3 are not available. Roughly speaking, the audiometric thresholds for our participants fell within the upper half of the reference distributions, and the mean audiograms were close to the reference audiograms corresponding to the 20th percentile (pink line).

Consistent with epidemiological data, the mean audiometric thresholds, averaged across ears for frequencies between 125 and 1500 Hz, increased (i.e., worsened) somewhat with increasing age group: They were 8.0, 9.6, and 13.3 dB HL for Groups O1, O2, and O3, respectively. A one-way between-subjects analysis of variance (ANOVA) revealed that the effect of age group was significant, *F*(2, 115) = 10.99, *p* < .001.

**Figure 2.**
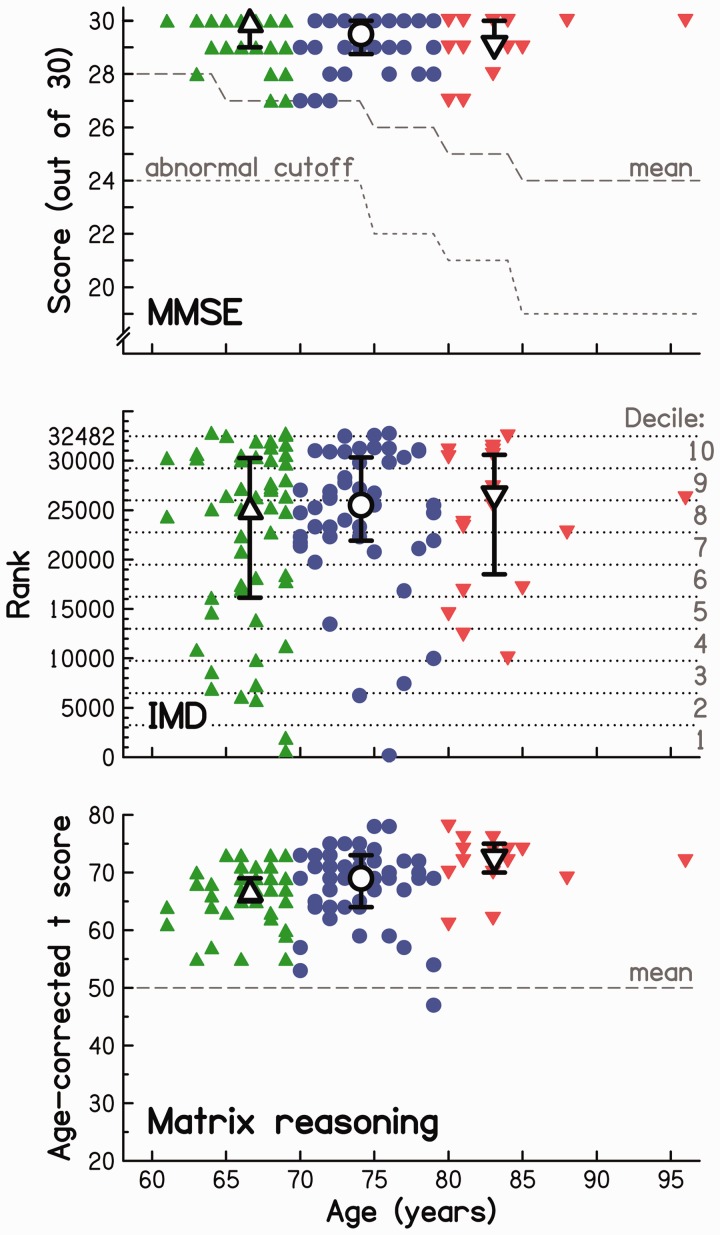
Individual (colored symbols) and age-group median (open symbols) cognitive and sociodemographic data as a function of participant age. Error bars represent the interquartile range. Top panel: MMSE scores along with reference mean data (Crum et al., 1993) and abnormal cutoff (Iverson, 1998) for a sample of 18,056 participants. Middle panel: IMD ranks; corresponding deprivation deciles are given on the right. Bottom panel: Age-corrected *t* scores (*M* = 50; *SD* = 10) for nonverbal fluid reasoning as measured by the Matrix Reasoning test. MMSE = Mini Mental State Examination; IMD = Index of Multiple Deprivation.

To ensure that the participants did not suffer from gross cognitive deficits that could have affected their ability to perform the TFS-AF test, the Mini Mental State Examination (MMSE; Folstein, Folstein, & McHugh, 1975) was administered. This test is extensively used by researchers and clinicians to measure cognitive impairment and to screen for dementia. The individual and median scores are given in the top panel of Figure 2 (due to a manipulation error, three MMSE scores were lost during the data analysis stage). They ranged from 27 to 30 (out of a maximum of 30) and were consistently at or above the age-dependent mean scores established by Crum, Anthony, Bassett, and Folstein (1993) for a large reference sample. Because scores in this range are considered to represent normal cognitive functioning (e.g., Bruce, Hoff, Jacobs, & Leaf, 1995), no potential participant was rejected. According to Shapiro–Wilk tests, scores were not normally distributed for any of the three age groups (all *p* < .001), and hence, the significance of group differences was assessed using a Kruskal–Wallis test. This yielded an H_(2)_ value of 2.627, which was not significant based on the χ^2^ distribution (*p* = .269; the H statistic follows a χ^2^ distribution when the number in each group is > 5).

**Figure 3.**
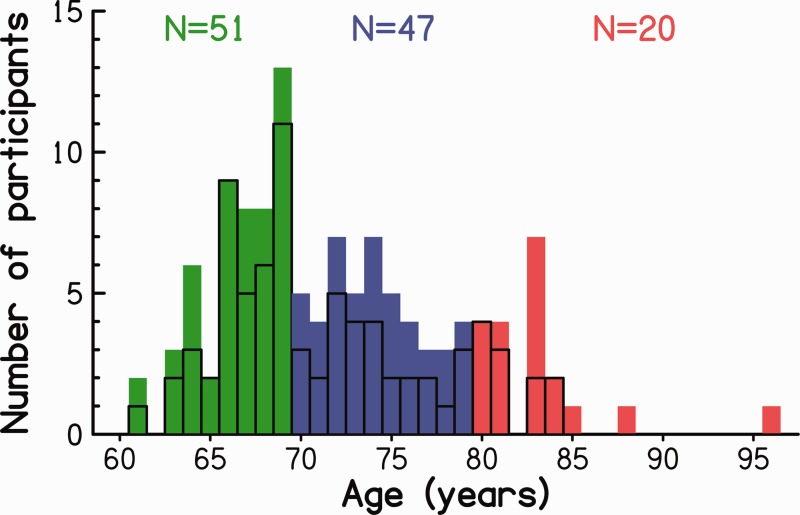
Age distribution of the 118 older participants for Groups O1 (green), O2 (blue), and O3 (red). The age distribution outlined by the heavy black line is for the female participants alone.

In total, there were 118 participants with ages from 61 to 96 years (*M* = 72 years, standard deviation [*SD*] = 6.5). Figure 3 shows the age distributions for the entire sample and for the female participants alone. Both distributions were positively skewed with frequent ages clustered toward the lower end of the age range. The sample contained almost twice as many female (*N* = 78) as male participants (*N* = 40). The percentage of female participants was 76, 60, and 55 for Groups O1, O2, and O3, respectively. The number of participants tested in Cambridge (47%) and in Nottingham (53%) was similar for each of the three age groups (O1: 45% vs. 55%; O2: 49% vs. 51%; O3: 45% vs. 55%).

To further characterize the sample and to evaluate the participants’ homogeneity across old adulthood in terms of their socioeconomic and cognitive status, additional demographic information and nonverbal fluid-reasoning scores were gathered for each participant.

First, the English Index of Multiple Deprivation (IMD; Smith et al., 2015a, 2015b) was determined for each participant. The IMD provides an overall *relative* measure of deprivation within a small area (or neighborhood) across England and corresponds to a weighted average of deprivation indices in seven domains: income, employment, health and disability, education skills and training, barriers to housing and services, living environment, and crime. England is divided into 32,844 Lower Level Super Output Areas (LSOAs), each with, on average, 1,500 residents or 650 households (Office for National Statistics, 2016). These LSOAs are ranked from most deprived (= 1) to least deprived (= 32,482) for each of the different domains of deprivation. The IMD for a given individual can be determined on the basis of the residential postcode of the person, which is linked to a LSOA (Ministry of Housing, Communities & Local Government, 2015). In contrast to individual-level measures of socioeconomic status (such as income, education, occupation), the IMD is a socioeconomic indicator at the area level. Consequently, comparing individuals based on their IMD might not be appropriate as, in every LSOA, individuals vary somewhat in deprivation. Here, the IMD is only used as a global descriptor of the study population, and the scores are not included in the statistical analyses conducted to predict TFS-AF scores. The distribution of IMD ranks for all participants is shown in the middle panel of Figure 2. While participants spanned the full range of IMD ranks, 64% fell into the three top deciles, indicating least deprivation. Twenty-six percent came from the middle four deciles, and only 9% from the lowest three deciles. The medians for the three age groups were similar and fell into the top half of the 8^th^ and bottom half of the 9^th^ deciles. The data violated both the assumption of homogeneity of variance, Levene’s test: *F*(2, 115) = 4.300, *p* = .016, and the assumption of normality (Shapiro–Wilk tests: all *p* ≤ .015). A Kruskal–Wallis test was used to assess the significance of differences between the three age groups. The test result, H_(2)_ = 0.662, was nonsignificant (*p* = .718). Hence, the age groups were similar in IMD.

Second, nonverbal fluid-reasoning abilities were assessed using the Matrix Reasoning (MR) test, taken from the Wechsler Abbreviated Scale of Intelligence (WASI; Wechsler, 1999), which is a standard measure of nonverbal intelligence in many test batteries of intelligence. The test contains 35 items presented in order of increasing difficulty. Each item is composed of a matrix of visual patterns with one element missing. The task is to choose from five response alternatives the one that completes the matrix. The two easiest items were used as practice. Following the test instructions, participants aged 45 to 79 years completed the first 32 items, while participants aged 80 years and above completed only the first 28. There was no time limit for completion of the test. Raw scores were transformed into age-corrected *t* scores (see Appendix A in Wechsler, 1999), with a mean of 50 and an *SD* of 10, to evaluate whether participants of different ages were sampled from the same cognitive stratum of the relevant underlying population. The data, shown in the bottom panel of Figure 2, indicated that almost all participants performed above average. The data violated both the assumption of homogeneity of variance, Levene’s test: *F*(2, 115) =3.746, *p* = .027, and the assumption of normality (Shapiro–Wilk tests: all *p* ≤ .016). A Kruskal–Wallis test revealed a significant difference in scores between the three age groups, H_(2)_ = 21.414, *p* < .001. Subsequent post hoc comparisons (two-tailed Mann–Whitney U test, uncorrected for multiple comparisons) confirmed significant differences between all groups (all *p* ≤ .032), with performance increasing with age group. This was confirmed by a moderately strong, significant Spearman correlation between age and age-corrected MR score (ρ = 0.38, *p* < .001). Taken together, these outcomes indicate that, relative to their underlying population, participants were more cognitively able (in terms of nonverbal fluid reasoning) as their age increased.

### TFS-AF Test

The TFS-AF test used a two-interval, two-alternative forced-choice procedure with visual feedback. On each trial, there were two consecutive intervals, separated by 500 ms. Each interval contained four consecutive 400-ms tones (including 20-ms raised-cosine rise/fall ramps), separated by 100 ms. In one interval, selected at random, the IPD of all tones was 0°. In the other interval, the 1^st^ and 3^rd^ tones were the same as in the standard interval while the 2^nd^ and 4^th^ tones differed in their IPD by 180°. Participants usually perceive pure tones with an IPD of 0° as being close to the center of the head, while low-frequency tones with an IPD of 180° are perceived as being lateralized toward one ear (Durlach & Colburn, 1978). Participants were asked to indicate (via either mouse clicks on virtual buttons displayed on a monitor or manual presses of buttons on a response box) which of the two intervals contained a sequence of tones that appeared to be more diffuse or to move within the head. They were asked to guess if they were unsure. The task is based on the assumption that sensitivity to IPD will be relatively good at low and medium frequencies, but will worsen at higher frequencies (Durlach & Colburn, 1978), and will approach zero above a participant-dependent frequency (Brughera et al., 2013; Hughes, 1940). The task is designed to determine the highest frequency for which IPD discrimination is possible, using a criterion of 71% correct (Levitt, 1971).

The initial frequency of the tones was usually set to 200 Hz. A few participants indicated that they could not hear any difference between the two intervals with this starting frequency, and for them, the run was aborted and the starting frequency was lowered to 100 Hz. The tone frequency was increased after two consecutive correct responses and decreased after one incorrect response. The frequency was changed by a factor of 1.4 until the first reversal occurred, then by a factor of 1.2 until the next reversal occurred, and by a factor of 1.1 thereafter. After eight reversals, the run was terminated, and the geometric mean of the values of the IPD at the last six reversals was taken as the threshold estimate. The lowest allowed frequency was 30 Hz. A run was considered as valid if the *SD* of the log values at the last six reversals was ≤ 0.2. When this value was exceeded, the threshold estimate was discarded, and an additional threshold run was conducted (this happened for 1% of all runs). Three valid threshold estimates were obtained and their geometric average computed. If the *SD* of the log values of the three estimates was > 0.2, an additional threshold run was conducted and the geometric mean computed for all four runs (this happened for 7% of the participants).

The presentation level in each ear for each test frequency was individually adjusted to 30 dB sensation level based on the measured audiometric thresholds; the required levels at intermediate frequencies were estimated by linear interpolation (in dB on a logarithmic frequency scale), or extrapolation when the frequency was below 125 Hz.

## Results

**Figure 4.**
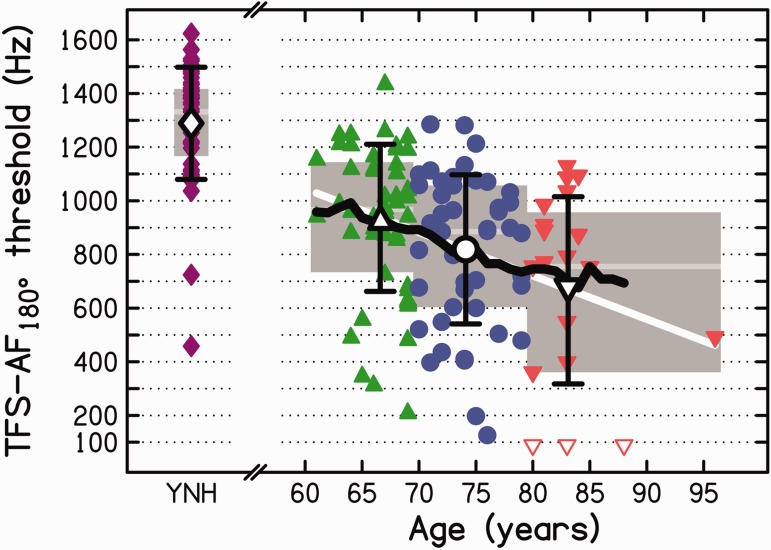
Colored filled symbols show the frequency at threshold for the TFS-AF test for the individual participants (open colored symbols denote participants who could not perform the task). Large open symbols show arithmetic mean thresholds with associated standard deviations (error bars) for the three older groups (O1, green; O2, blue; O3, red) from the present study and for 49 YNH listeners (<30 years, purple) tested in other studies. Means for the older age groups are plotted at the arithmetic mean age of the group members. The gray-shaded areas and gray horizontal bars represent the interquartile range (IQR) and median for each age group. The thick black line shows the running average, computed as the arithmetic mean within a 9-year time window symmetrically centered on each full-year age between 61 and 88 years (excluding the threshold for the oldest participant). The thick white line is a regression line fitted to the individual thresholds for the three older groups. *Note*. TFS-AF = temporal fine structure-adaptive frequency.

In our previous studies using the TFS-AF test, the thresholds were plotted on a logarithmic frequency scale. However, for the present data, the distribution of thresholds within a given age range was more normal when plotted on a linear scale than when plotted on a logarithmic scale. Hence, we decided to plot and analyze the thresholds on a linear frequency scale. Figure 4 shows the results of the TFS-AF test as a function of age. Individual thresholds are plotted as filled colored symbols. All participants were able to complete the TFS-AF test except for three from Group O3, who reported that they could not hear any difference between the two intervals. They abandoned the test before the end of the adaptive track even though they were instructed to guess if they were unsure. For display of the data in Figure 4 and for subsequent statistical analyses, the thresholds for the “nonperformers” (shown as open symbols) were set to the mean threshold that would be expected by random guessing. To determine this, we simulated 10,000 runs of the task using a starting frequency of 200 Hz. The mean threshold was 80 Hz, and the median was 55 Hz.

To assess the test–retest reliability of the TFS-AF test, the three threshold estimates were entered into an intraclass correlation (ICC) analysis, using a two-way mixed-effects model and an absolute-agreement definition (ICC_(A,k)_; McGraw & Wong, 1996). Because no graded measure could be obtained for the three “nonperformers,” results for these were not included in the analysis. Reliability was “excellent” (Cicchetti, 1994), with the average-measures ICC_(A,3)_ being 0.879 [for Groups O1, O2, and, O3, the ICC_(A,3)_ was 0.861, 0.888, and 0.863, respectively] and the associated 95% confidence interval (CI) ranging from 0.835 to 0.913, *F*(114, 228) = 8.199, *p* < .001.

The individual TFS-AF thresholds for the older participants did not exceed 1500 Hz, and their overall arithmetic mean threshold was 844 Hz (*SD* = 303). Both the highest (best) and the lowest (worst) thresholds decreased with increasing age group.

For comparison, the left part of Figure 4 shows TFS-AF thresholds for 49 young (*M* = 21 years, range = 18 to 30) adults with audiometric thresholds ≤20 dB HL between 125 and 8000 Hz in both ears (purple symbols), tested as part of other studies (Füllgrabe et al., 2017, plus unpublished results from our laboratory). The arithmetic mean threshold for the YNH group was 1289 Hz (*SD* = 209). Two YNH participants gave noticeably lower thresholds than the rest of their age group; however, in contrast to Group O3, all young participants were able to complete the TFS-AF test.

The ICC_(A,3)_ for the YNH participants was 0.821, and the associated 95% CI ranged from 0.713 to 0.893, *F*(48, 96) = 5.624, *p* < .001, indicating “good” to “excellent” test–retest reliability.

In agreement with previous studies assessing binaural TFS sensitivity for participants with normal audiometric thresholds in the low-frequency range (Füllgrabe, 2013; Füllgrabe et al., 2015; Grose & Mamo, 2010; Hopkins & Moore, 2011; Moore, Vickers, et al., 2012; Ross, Fujioka, et al., 2007), age-group mean thresholds (large open symbols in Figure 4) worsened from young to old adulthood. Because the data for the YNH listeners were not normally distributed (Shapiro–Wilk test, *p* ≤ .001), the difference in TFS-AF thresholds between the YNH group and the entire group of older listeners was assessed using a Mann–Whitney U test. The result was highly significant (*p* < .001; one-tailed).

More important, our data reveal that binaural TFS sensitivity also declines across age groups during older adulthood for participants with normal audiometric thresholds at low frequencies. Mean threshold was 937, 819, and 666 Hz for Groups O1, O2, and O3, respectively. A one-way between-subjects ANOVA on the individual thresholds for the older listeners showed a significant main effect of age group, *F*(2, 115) = 6.562, *p* = .002. Subsequent one-tailed least-significant difference post hoc tests indicated that TFS-AF thresholds differed significantly between Group O1 and each of Groups O2 and O3 (*p* = .024 and < .001, respectively) and also between Groups O2 and O3 (*p* = .025).

The individual TFS-AF thresholds were significantly correlated with age (*r* = .35, *p* < .001, one-tailed), and the correlation remained significant when the effect of the pure-tone average at low frequencies (PTA_125–1500 Hz_) was partialled out (*r* = .27, *p* < .002, one-tailed). To characterize and predict the change in TFS-AF threshold throughout older adulthood, a linear regression line was fitted to the individual TFS-AF thresholds (see thick white line in Figure 4), giving the following fit:

TFS-AFthreshold(Hz)=(-16.2×Age)+2016(1)

where the constant −16.2 has units Hz/year, and the constant 2016 has units Hz. The slope of the regression line was significantly different from 0, *t*_(116)_ = −4.013, *p* < .001. This indicates that, on average, the highest frequency up to which binaural TFS information can be processed declines by about 162 Hz for every 10-year increase over the age range 60 to 85 years. However, the model accounted only for 12% of the variance in TFS-AF thresholds, indicating that factors other than age contribute to IPD discrimination. Possible factors are audiometric threshold and cognition. All of the outcomes discussed earlier were hardly changed when the threshold for the 96-year-old participant was excluded from the analysis.

In addition to the observed decline in binaural TFS sensitivity with increasing age, within-group variability in thresholds increased with age group, when expressed as the *SD* (209, 274, 278, and 349 Hz) or the coefficient of variation [that is, (*SD*/mean) × 100; 16%, 29%, 34%, and 52%] for groups YNH, O1, O2, and O3, respectively. Including all four age groups, a comparison of the coefficients of variation across groups, using Levene’s *F* test, indicated a significant effect, *F*(3, 163) = 17.470, *p* < .001. Subsequent uncorrected one-tailed *t* tests revealed that there were significant differences between the YNH group and each of the three older groups (all *p* < .001) as well as among the older groups (both *p* ≤ .003); only Groups O1 and O2 did not differ significantly (*p* = .08).

To study other potential contributors to the individual variability in TFS-AF thresholds, correlations were calculated between TFS-AF thresholds and general cognitive functioning (based on age-corrected scores for the MR test) and the PTA across ears for the low-frequency region (PTA_125–1500 Hz_) and the high-frequency region (PTA_4000–8000 Hz_). Because only the PTA_125–1500 Hz_ values were normally distributed, Spearman correlations were used. The results and associated significance levels are shown in Table 1. TFS-AF thresholds were weakly but significantly correlated with PTA_125–1500 Hz_ values (ρ = −0.26, *p* = .005, two-tailed). This contrasts with the lack of significant effect reported for studies using smaller samples of older listeners with normal or near-normal low-frequency hearing (Füllgrabe, 2013; Füllgrabe et al., 2015; Moore, Glasberg, et al., 2012; Neher et al., 2012) but is consistent with results from studies using participants who were selected to have a wide range of audiometric thresholds at low frequencies (Füllgrabe & Moore, 2017; King, Hopkins, & Plack, 2014; Moore & Sęk, 2016). Neither high-frequency hearing sensitivity (ρ = −0.08, *p* = .382, two-tailed) nor age-corrected MR scores (ρ = 0.10, *p* = .272, two-tailed) were significantly associated with TFS-AF thresholds. However, when the raw scores from the MR task were used (i.e., without controlling for the effect of age on cognitive performance) for all older participants who completed the first 32 items (i.e., the 98 participants aged 61 to 79 years), the correlation with TFS-AF thresholds was weak but significant (ρ = 0.346, *p* < .001).

**Table 1.** Nonparametric Correlation Coefficients (Spearman’s ρ With Associated Two-Tailed Significance Levels in Parentheses) Between Individual TFS-AF Thresholds (Hz), Age-Corrected *t* Scores for the Matrix Reasoning Test, and Low-Frequency (PTA_125–1500 Hz_) and High-Frequency (PTA_4000–8000 Hz_) Pure-Tone Averages for the 118 Older Participants.

	Matrix Reasoning	PTA_125–1500 Hz_	PTA_4000–8000 Hz_
TFS-AF threshold	0.102 (0.272)	−0.255 (0.005)	−0.081 (0.382)
Matrix Reasoning		0.174 (0.059)	0.219 (0.017)
PTA_125–1500 Hz_			0.469 (<0.001)

*Note*. PTA = pure-tone average; TFS-AF = temporal fine structure-adaptive frequency.

## Discussion

The aim of this study was to characterize changes in the processing of binaural TFS information during older adulthood, an age range where speech perception—especially in noisy environments—becomes more difficult, and to provide representative data for researchers and clinicians who wish to assess binaural TFS sensitivity.

### Influence of Age and Hearing Loss on the Processing of Binaural TFS Information

The results for our large sample of older listeners with normal to near-normal low-frequency audiometric thresholds revealed that binaural TFS sensitivity declines with increasing age; on average, TFS-AF thresholds worsened by 162 Hz for every 10-year increase in age between 60 and 85 years. Individual variability also increased with increasing age, as already noticed in smaller studies assessing the highest frequency at which a change in IPD could be detected by young, middle-aged, and normal-hearing older listeners (Grose & Mamo, 2010; Ross, Fujioka, et al., 2007).

Using the regression model fitted to the data for the older participants (Equation 1), the mean TFS-AF threshold for the YNH listeners was predicted to be reached for an age of 45 years. This is consistent with the idea that adult binaural TFS sensitivity remains roughly constant until the middle of the fourth decade of life, but declines thereafter. Alternatively, the decline in binaural TFS sensitivity between young adulthood and about 60 years of age may follow a shallower trajectory than during older adulthood. At present, neither the findings of the present study nor other published data (Füllgrabe, 2013; Grose & Mamo, 2010; Ross, Fujioka, et al., 2007) allow a decision as to which of these alternatives is more accurate.

We also found that TFS-AF thresholds tended to decline with increasing audiometric threshold at low frequencies, even though all participants had normal to near-normal hearing over this frequency range. While some previous studies of binaural TFS processing failed to show an association with audiometric thresholds when the latter were in the near-normal range (Füllgrabe et al., 2015; Moore & Sęk, 2016; Neher et al., 2012), this may have been a consequence of a lack of statistical power. It seems that the definition of a “normal” range of thresholds is arbitrary and that performance on some tasks worsens even when audiometric thresholds are only slightly above 0 dB HL (Bernstein & Trahiotis, 2016; Léger, Moore, & Lorenzi, 2012).

The present results confirm earlier studies (e.g., Füllgrabe & Moore, 2017; Moore, Glasberg, et al., 2012) showing that there is no association between binaural TFS sensitivity and audiometric thresholds at high frequencies, indicating that high-frequency hearing loss is not a marker of changes in TFS sensitivity, as has been speculated previously (Smoski & Trahiotis, 1986; Strelcyk & Dau, 2009).

It has been suggested that increasing age has a stronger deleterious effect on binaural TFS sensitivity than hearing loss (Füllgrabe & Moore, 2017; Moore, 2016), based on the observation of larger group differences in binaural TFS sensitivity between YNH and ONH listeners than between ONH and OHI listeners (e.g., Hopkins & Moore, 2011), and higher correlations between binaural TFS thresholds and age than between TFS thresholds and low-frequency audiometric thresholds (Füllgrabe, 2013; Füllgrabe et al., 2015; Moore, Glasberg, et al., 2012). However, comparison of the size of the effects of age and low-frequency audiometric thresholds is problematic, as the measured effects and correlations will depend on the populations studied, specifically on the age ranges and audiometric threshold ranges of the tested sample. A study of binaural sensitivity for a representative sample of the adult population as a whole would be needed to establish the relative importance of age and audiometric thresholds. An alternative approach is to perform a meta-analysis of existing data sets including participants with a wide range of ages and audiometric thresholds (Füllgrabe & Moore, in press).

### Methodological Considerations

Previous studies have often neglected the nonaudiometric characteristics of their participants (such as their socioeconomic and cognitive status). This may have led to participant samples that differed across the age ranges investigated and were unrepresentative of the general population. In the present study, additional demographic and cognitive information was gathered to assess the homogeneity of the participants across the older age groups and to allow a direct comparison with the patient population seen in audiology clinics. The age groups in the present study were broadly similar to each other in terms of their socioeconomic background and cognitive status as measured by the MR test. However, the IMD ranks and age-corrected MR scores fell toward the upper end of the normal range, indicating less deprivation and higher nonverbal fluid-reasoning abilities in our sample than in the general population. This was somewhat unexpected, as the study was conducted in two cities with historically different socioeconomic backgrounds, and the recruitment process was designed to reach volunteers with diverse socioeconomic backgrounds and cognitive abilities. While raw performance in the MR test declined with age, age-corrected performance actually increased with age group in our sample of ONH listeners. This might be the result of a sampling bias due to the use of an audiometric inclusion criterion for low frequencies. If hearing and cognitive status are both linked to the same underlying variable (such as cardiovascular status or inflammatory factors; e.g., Chung et al., 2009), then the recruitment of audiometrically normal to near-normal-hearing participants would have led to the selection of good cognitive performers. As the fixed audiometric inclusion criterion becomes more stringent with increasing age of the possible candidate (i.e., fewer possible people are eligible), the sample is more and more biased toward cognitive high-performers relative to the underlying stratum.

How representative our sample is of the average patient attending audiology services remains to be established, as currently only some demographic information about such patients, such as age, but not socioeconomic and cognitive background, is gathered (e.g., Hind et al., 2011). However, our results suggest that (age-independent) variability in nonverbal fluid reasoning does not have a marked influence on TFS-AF thresholds, as there was no significant correlation between TFS-AF thresholds and age-corrected MR scores. This is consistent with the finding of Füllgrabe et al. (2015) that within a group of older listeners with a narrower age range (60 to 79 years), MR scores were not significantly correlated with a composite measure of sensitivity to TFS, although in that study, raw (i.e., not age-corrected) MR scores were used, and the sample size was much smaller than in the present study. However, Füllgrabe et al. (2015) did show that within their older group, there was a correlation between composite sensitivity to TFS and performance on several other cognitive tests (such as the Digit Span test, some of the subtests of the Test of Everyday Attention, part B of the Trail Making test, and the Block Design test). Thus, the influence of cognition on performance of tests of TFS sensitivity remains somewhat unclear and is likely to be complex.

Although ARHL is usually characterized by greater audiometric losses at high than at low frequencies, our use of older participants with normal or near-normal low-frequency hearing might have resulted in an overestimation of the binaural TFS sensitivity that would occur for an age-matched population with more variability in low-frequency hearing sensitivity. For the general population, we would expect this effect to be small because low-frequency hearing loss is relatively rare among older people. For example, in the United Kingdom, only 20% of people aged 61 to 70 years have low-frequency audiometric thresholds (averaged over 250, 500, and 1000 Hz) greater than 25 dB HL (Davis, 1995). However, for older people attending an audiology clinic, the effect might be much larger. For example, Bisgaard, Vlaming, and Dahlquist (2010) showed that the majority of visitors to a Swedish audiology clinic had low-frequency losses greater than 25 dB HL.

In behavioral studies, part of any observed effect of age on hearing may have been caused by nonauditory factors. While the lack of practice effects suggests that the TFS-AF task is easy to perform even for older participants (Füllgrabe et al., 2017; Füllgrabe & Moore, 2017), age-related changes in factors such as processing efficiency (the ability to make use of sensory information) could have contributed to the worsening in performance with increasing age. For example, Wallaert et al. (2016) showed that poorer amplitude-modulation (AM) detection for older than for young participants, as found in their study and by Füllgrabe et al. (2015), could be modeled by increased internal noise for the former group. Whiteford, Kreft, and Oxenham (2017) showed that thresholds for detecting frequency modulation (FM) were correlated with age for low-rate but not high-rate FM, but this correlation was found only when the threshold for detecting AM was controlled for. Thresholds for detecting AM and FM were correlated even for low FM rates, at which AM and FM are thought to be detected using different neural codes (Moore & Sęk, 1996). Whiteford et al. (2017) argued that this might reflect an influence of more central factors, such as sustained attention. However, the results of a study of Moore, Heinz, Braida, and Leger (2018) suggest that the influence of nonsensory factors is small. They assessed sensitivity to ITD in the ENV_p_ and TFS_p_ of AM tones for young and older participants, all with normal hearing at low frequencies. The older participants performed only slightly more poorly than the young participants (by a mean factor of 1.16) for discrimination of ITD in ENV_p_, but performed markedly more poorly (by a mean factor of 1.74) for discrimination of ITD in TFS_p_, suggesting a selective deficit in the binaural processing of TFS, with at most a small role of nonauditory factors. King et al. (2014) also found smaller effects of age for discrimination of ITDs in the ENV_p_ than in the TFS_p_ of AM stimuli.

### Possible Mechanisms Underlying the Effect of Age

Performance on tests of binaural TFS sensitivity, like the TFS-LF and TFS-AF tests, may depend partly on the monaural coding of TFS information prior to binaural interaction (Füllgrabe et al., 2017; Whiteford et al., 2017). Consistent with this idea, scores for tests of monaural and binaural TFS sensitivity are correlated, but not highly so (Füllgrabe et al., 2015; Moore, Vickers, et al., 2012). While, in principle, the moderate correlation might be partly caused by an age-related decline in processing efficiency, the data reviewed in the Methodological Considerations section suggest that the influence of changes in processing efficiency is likely to be small.

Age-dependent changes that might affect the processing of monaural TFS information (prior to the point in the auditory pathway where binaural interaction occurs) include the following:

1. Loss of inner hair cells, synapses, or auditory neurons, which would lead to more “noisy” TFS_n_ (Makary, Shin, Kujawa, Liberman, & Merchant, 2011; Schuknecht, 1993; Sergeyenko et al., 2013);
2. Less effective enhancement of the precision of temporal coding resulting from convergence of neural inputs in the cochlear nucleus and high centers in the brain stem (Joris, Carney, Smith, & Yin, 1994).

Age-dependent changes that might affect the processing of either or both monaural and binaural TFS information include the following:

1. Loss of myelin sheaths around neurons in central auditory pathways, which increases the temporal “jitter” in neural conduction times (Bartzokis, 2004);
2. Loss of inhibition, which can disrupt the mechanisms involved in decoding TFS_n_ information (Caspary, Raza, Lawhorn Armour, Pippin, & Arneric, 1990; Gleich, Hamann, Klump, Kittel, & Strutz, 2003; Shamma, 1985).

### Applications and Recommendations

At present, there is no treatment for reduced sensitivity to binaural TFS, although it is possible that some of the underlying factors might be amenable to treatment (e.g., synaptopathy; Liberman & Kujawa, 2017). Tests like the TFS-AF test might be useful in detecting early signs of hearing damage, like synaptopathy, which are not revealed by the audiogram, because any treatment is likely to be more effective if administered before the deficit becomes too severe.

The TFS-AF test may also be useful, in addition to pure-tone audiometry, as part of an audiological assessment for the purpose of the diagnosis or management of hearing difficulties. One possible use of such tests is in predicting the difficulties in speech perception that might be experienced in everyday life. Füllgrabe et al. (2015) showed that the ability to understand speech in background sounds was correlated with a composite measure of sensitivity to TFS for listeners with audiometrically normal hearing. If the main problem of an HI person is loss of sensitivity to TFS rather than reduced audibility, then simple amplification via HAs may be of limited benefit, although other aspects of HA processing may help (Neher, Wagener, & Latzel, 2017).

It would also be interesting to know whether people with unusually low (poor) TFS-AF thresholds, such as two of the YNH participants, have particular difficulties in understanding speech in the presence of spatially distributed competing sounds. While several studies have addressed the question of how much of the observed variability in speech perception of HI listeners can be explained by individual differences in TFS sensitivity, most of these studies have used relatively small samples (e.g., Füllgrabe et al., 2015; Hopkins & Moore, 2011; Neher et al., 2012; Strelcyk & Dau, 2009). Larger scale studies with more carefully controlled listener groups in terms of age, hearing loss, and cognitive ability are warranted to test the predictive power and, thus, the clinical usefulness of suprathreshold tests, such as the TFS-AF test.

Another, potentially promising use of the TFS-AF test is in selecting appropriate signal processing for binaurally fitted HAs. For example, binaural beamforming HAs can selectively amplify sounds from a specific direction but at the expense of discarding IPD and interaural level cues (Launer, Zakis, & Moore, 2016). Such systems might be beneficial for listeners with poor binaural TFS sensitivity, for whom the loss of IPD cues would not be a major disadvantage. However, listeners with good sensitivity to IPD cues might suffer more from the loss of IPD cues, so binaural beamforming might be less appropriate for such listeners (Neher et al., 2017). They might instead benefit from processing that uses IPD cues to enhance interaural level differences (Moore, Kolarik, Stone, & Lee, 2016).

For researchers and clinicians who wish to broaden the characterization of their participants and patients, the TFS-AF test seems to be a suitable candidate for the evaluation of binaural TFS sensitivity, for the following reasons: (a) it can be performed by most people and hence yields a graded measure of binaural TFS sensitivity, independently of age and hearing status, (b) it does not require practice to achieve stable threshold estimates, and (c) its administration time is relatively short (Füllgrabe et al., 2017; Füllgrabe & Moore, 2017). These attributes might make the TFS-AF test a better choice for a clinical evaluation of binaural TFS sensitivity than other tests that have been frequently used. For example, the TFS-LF test does not yield a graded measure of sensitivity for all listeners, and the binaural masking level difference depends partly on the use of energy and ENV cues (Mao, Koch, Doherty, & Carney, 2015) and is prone to large training effects (Hafter & Carrier, 1970).

To make the TFS-AF test usable for a wide range of listeners, we recommend a relatively large value for the fixed change in IPD (e.g., 180°) to ensure that the task is as easy as possible for all listeners at the start of the threshold run; the potential ambiguity of this antiphase condition as to whether the sound is leading in time at the left or the right ear does not negatively affect performance (Füllgrabe et al., 2017; Füllgrabe & Moore, 2017). Also, using a low sensation level of 30 dB has the advantage of avoiding uncomfortable loudness for listeners with mild-to-moderate hearing loss at low frequencies while being high enough to lead to asymptotic performance (Hopkins & Moore, 2010). Füllgrabe and Moore (2017) estimated that three threshold runs should be conducted to achieve a reliable measure, even for older listeners. Here, we found that three runs yielded a high ICC_(A,3)_ of 0.879. Three runs would take about 18 min (5 min per run, with a short break between runs; Füllgrabe & Moore, 2017). In view of the time restrictions of an audiological evaluation, we recomputed the test–retest reliability based on the first two estimates (out of the three obtained) and found still “excellent” reliability, with an ICC_(A,2)_ of 0.827 [the associated 95% CI ranged from 0.751 to 0.880; *F*(115, 115) = 5.761, *p* < .001]. This indicates that only two TFS-AF threshold estimates can provide a reliable evaluation of binaural TFS sensitivity, which would take about 11 min.

## Conclusions

Binaural sensitivity to TFS, assessed using the TFS-AF test, showed a significant decline (worsening) with increasing age over the older age range investigated here (61–96 years). The mean threshold decreased by 162 Hz for each 10-year increase in age between the ages of 60 and 85 years; the data for ages above 85 years were deemed too sparse to obtain a reliable estimate. There was, however, a large range of TFS-AF thresholds for participants with similar ages. The within-group variability increased from the youngest to the oldest groups. TFS-AF thresholds also showed a significant decline as the average audiometric threshold at low frequencies worsened.

The participants in our sample showed less deprivation and higher nonverbal intelligence than for the general population. However, there was no significant correlation between TFS-AF thresholds and age-corrected performance on the MR test. This suggests that age-unrelated variability in nonverbal fluid reasoning does not have a marked influence on TFS-AF thresholds.
